# Interactions between *Balantidium ctenopharyngodoni* and microbiota reveal its low pathogenicity in the hindgut of grass carp

**DOI:** 10.1186/s12866-023-03154-8

**Published:** 2024-01-03

**Authors:** Weishan Zhao, Xialian Bu, Weitian Zhou, Qingwen Zeng, Tian Qin, Shangong Wu, Wenxiang Li, Hong Zou, Ming Li, Guitang Wang

**Affiliations:** 1 Institute of Hydrobiology, Chinese Academy of Sciences, Wuhan, 430072 China; 2 Key Laboratory of Breeding Biotechnology and Sustainable Aquaculture, Institute of Hydrobiology, Chinese Academy of Sciences, Wuhan, 430072 China; 3 University of Chinese Academy of Sciences, Beijing, 100049 China

**Keywords:** *Balantidium ctenopharyngodoni*, Grass carp, Intestinal microbiota, Metabolites, Ciliate

## Abstract

**Background:**

Hosts, parasites, and microbiota interact with each other, forming a complex ecosystem. Alterations to the microbial structure have been observed in various enteric parasitic infections (e.g. parasitic protists and helminths). Interestingly, some parasites are associated with healthy gut microbiota linked to the intestinal eubiosis state. So the changes in bacteria and metabolites induced by parasite infection may offer benefits to the host, including protection from other parasitesand promotion of intestinal health. The only ciliate known to inhabit the hindgut of grass carp, *Balantidium ctenopharyngodoni*, does not cause obvious damage to the intestinal mucosa. To date, its impact on intestinal microbiota composition remains unknown. In this study, we investigated the microbial composition in the hindgut of grass carp infected with *B. ctenopharyngodoni*, as well as the changes of metabolites in intestinal contents resulting from infection.

**Results:**

Colonization by *B. ctenopharyngodoni* was associated with an increase in bacterial diversity, a higher relative abundance of *Clostridium*, and a lower abundance of Enterobacteriaceae. The family Aeromonadaceae and the genus *Citrobacter* had significantly lower relative abundance in infected fish. Additionally, grass carp infected with *B. ctenopharyngodoni* exhibited a significant increase in creatine content in the hindgut. This suggested that the presence of *B. ctenopharyngodoni* may improve intestinal health through changes in microbiota and metabolites.

**Conclusions:**

We found that grass carp infected with *B. ctenopharyngodoni* exhibit a healthy microbiota with an increased bacterial diversity. The results suggested that *B. ctenopharyngodoni* reshaped the composition of hindgut microbiota similarly to other protists with low pathogenicity. The shifts in the microbiota and metabolites during the colonization and proliferation of *B. ctenopharyngodoni* indicated that it may provide positive effects in the hindgut of grass carp.

**Supplementary Information:**

The online version contains supplementary material available at 10.1186/s12866-023-03154-8.

## Background

The intestine is colonized by a huge number of microorganisms, playing an important role in health and disease [1]. These microorganisms have been well adapted to the intestinal mucosa, contributing to the host’s nutrition, metabolism and immune functions [2, 3]. Thus the gut microbiota has been characterized as a microbial organ tightly associated with the host’s health [1, 4].

Grass carp is native to rivers and lakes in eastern Asia, with a wide distribution in China [5]. As an economically important freshwater fish and aquatic plant consumer, it was introduced to many countries for the purpose of human consumption and biological control of aquatic weeds [6, 7]. Microbiota establishes themselves in the intestines of fish larvae, and their diversity increases as the fish grows [8–10]. More than ten years ago, it was proposed that the core microbiota in grass carp are composed of Proteobacteria, Firmicutes, and Actinobacteria [11]. However, the microbial composition varies between individuals of the same species, and even within the same individual at different times [8]. Moreover, diets, antibiotics, immune deficiency, environmental factors or infections may lead to variations in bacterial diversity [8, 12]. Shifts in the microbial composition may allow aggravating factors to amplify changes in specific bacterial species, resulting in intestinal dysbiosis, which is a disruption of the symbiotic relationship between microbial communities and hosts [13].

Furthermore, specific intestinal microbial patterns are known to be related to the colonization with parasitic protozoa [14, 15]. Although the mechanisms underlying the association between protozoan infections and microbial variations are still poorly understood [16], the complex interactions among the host, parasite, and host’s intestinal microbiota have important functional implications for the outcomes of parasitic infections and diseases. Long-term colonization of some protists indicates that they may play alternative roles in the intestine, other than directly affecting host health [17, 18]. For example, increased bacterial diversity or higher abundance of probiotics was found in association with the presence of commensal protozoa (e.g. low-pathogenic *Entamoeba* and *Blastocystis*).

*Balantidium ctenopharyngodoni* is an obligate intestinal ciliate inhabiting the hindgut of grass carp, with high prevalence, unique infection features, and asymptomatic carrier status [19, 20]. An in vitro cultivation method of *B. ctenopharyngodoni* has been explored using a strain of intestinal bacterium [20, 21]. However, the mechanism of interaction between *B. ctenopharyngodoni* and the intestinal microbiota remains unknown. Additionally, the pathogenicity of *B. ctenopharyngodoni* is still unclear. In this study, we aim to investigate the associations between *B. ctenopharyngodoni* and microbiota, the relationships between the infection intensity of *B. ctenopharyngodoni* and the relative abundance of specific microbial taxa, as well as the changes of metabolites in the hindgut affected by the infection with *B. ctenopharyngodoni*. Our results indicated that infection with *B. ctenopharyngodoni* may increase the diversity of microbiota, but decrease the relative abundance of Enterobacteriaceae and Aeromonadaceae. Moreover, it was associated with a high content of creatine in the hindgut. Our findings offer valuable insights into how *B. ctenopharyngodoni* affects the intestinal health of grass carp. These may further give explanations on why *B. ctenopharyngodoni* is able to form a stable relationship with its host. Thus, we inferred that the presence of *B. ctenopharyngodoni* is closely related to a healthy gut microbiota and that it may play a beneficial role in grass carp through modulating intestinal bacteria.

## Results

### Comparison of bacterial composition and diversity in the hindgut of grass carp

We obtained 2,393,030 high-quality 16 S rRNA sequences from 15 samples of grass carp hindgut contents. A sampling depth of 45,000 reads was resampled according to the rarefaction curves (Fig. S1).

**Fig. 1:**
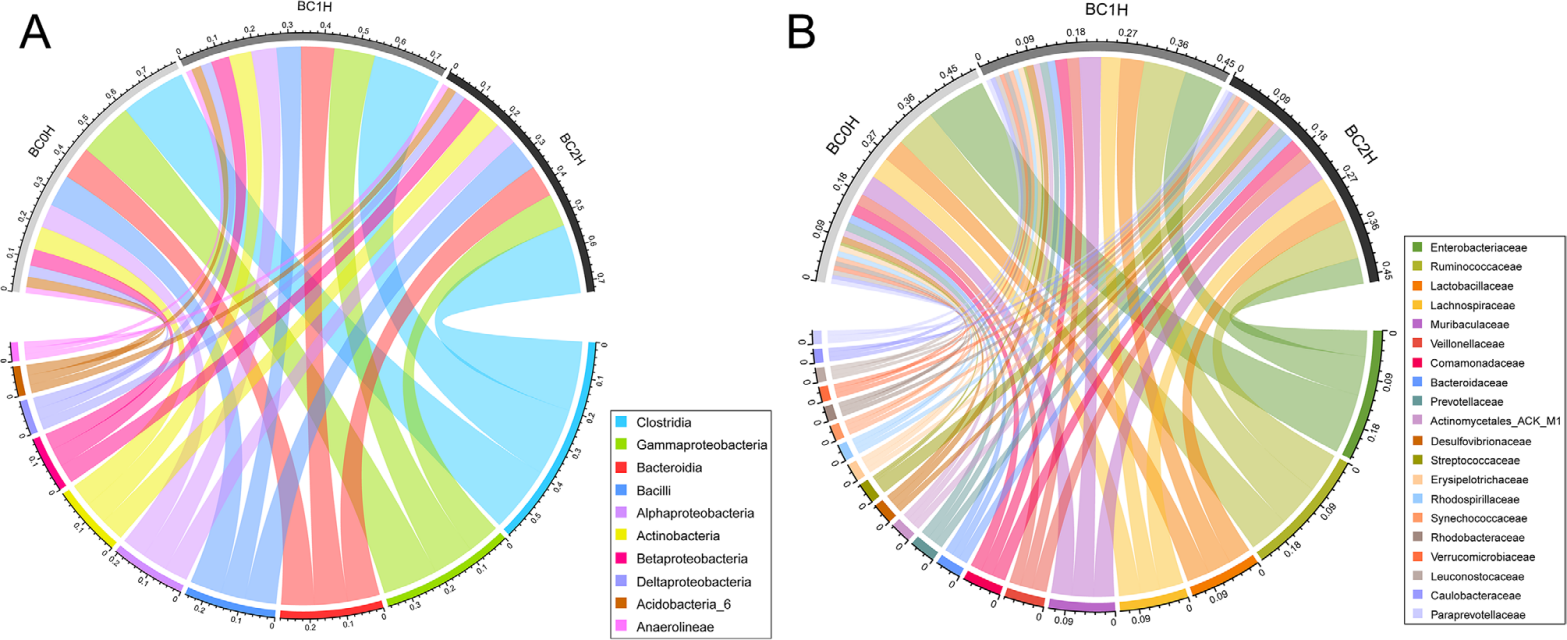
Bacterial composition in the hindgut of grass carp at the class level (**A**, top 10 classes) and family level (**B**, top 20 families). BC0H represents a group not infected with *B. ctenopharyngodoni*. BC1H represents a group infected with a low abundance of *B. ctenopharyngodoni*. BC2H represents a group infected with a high abundance of *B. ctenopharyngodoni*

**Fig. 2:**
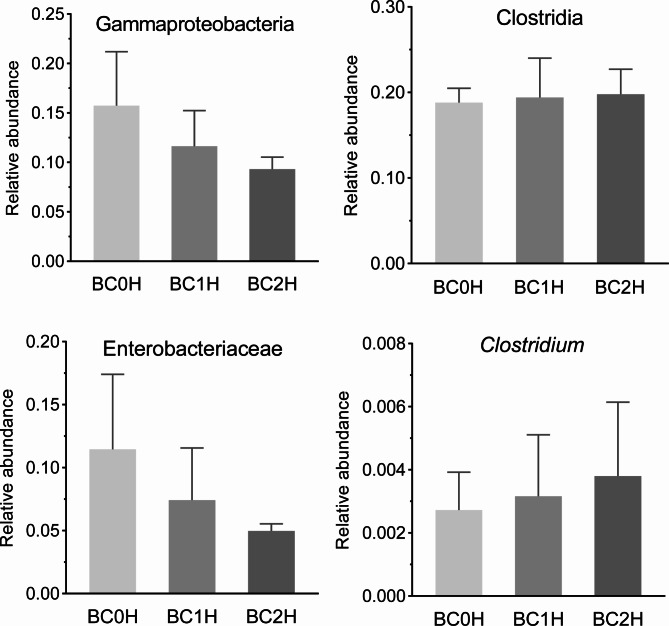
The relative abundance of specific taxa in the hindgut of three groups of grass carp

At the class level, 129 classes were shared by all samples, in which the top three classes in terms of relative abundance were Clostridia (BC0H (fish uninfected with *B. ctenopharyngodoni*): 18.80%±1.68%; BC1H (fish with a low-intensity infection): 19.42%±4.60%; BC2H (fish with a high-intensity infection): 19.78%±2.92%), Gammaproteobacteria (BC0H: 15.74%±5.46%; BC1H: 11.63%±3.60%; BC2H: 9.33%±1.20%) and Bacteroidia (BC0H: 9.39%±1.74%; BC1H: 9.49%±1.83%; BC2H: 9.24%±1.73%) (Fig. 1A). Compared to the BC0H group, Gammaproteobacteria had a low relative abundance in infected groups (BC1H, BC2H), while the relative abundance of Clostridia slightly increased in the infected groups (Fig. 2). At the family level, Enterobacteriaceae (BC0H: 11.45%±5.96%; BC1H: 7.43%±4.13%; BC2H: 4.97%±0.57%) and Ruminococcaceae (BC0H: 7.62%±1.64%; BC1H: 8.14%±4.23%; BC2H: 7.59%±2.21%) were the predominant families in all three groups, and the relative abundance of Enterobacteriaceae was lower in the infected groups (Figs. 1B and 2). Our results also showed that the hindgut of grass carp possesses a core microbiota, and the relative abundance of Firmicutes increased with the intensity of infection of *B. ctenopharyngodoni*, while the relative abundance of Proteobacteria decreased (Fig. S2).

The alpha diversity, species richness (ACE and Chao1), and species diversity (Shannon, Simpson) were higher in infected groups than in the BC0H group (Table S1). Moreover, the BC2H group had more unique classes (10, Fig. S3A) and families (44, Fig. S3B). Permutational multivariate analysis of variance (PERMANOVA) showed that there was no significant difference in the composition of bacterial communities among BC0H, BC1H and BC2H groups (BC0H-BC1H, *p* = 0.800; BC0H-BC2H, *p* = 0.326; BC1H-BC2H, *p* = 0.587). Principal coordinates analysis (PCoA) showed that bacterial communities in the three groups were not clearly distinguishable (Fig. S4).

A total of 28 prokaryotic clades were screened out with a linear discriminant analysis (LDA) threshold score of 2.5 (Fig. S5). Microbial taxa with significantly higher abundance in the BC2H group mainly belonged to the families Fusobacteriaceae (genus u114), Gemmatimonadaceae (genus *Gemmatimonas*), Halomonadaceae, and Sphingobacteriaceae. With regard to the BC0H group, the families Enterobacteriaceae and Aeromonadaceae had significantly higher relative abundance.

### Significant changes in predicted bacterial functions

**Fig. 3:**
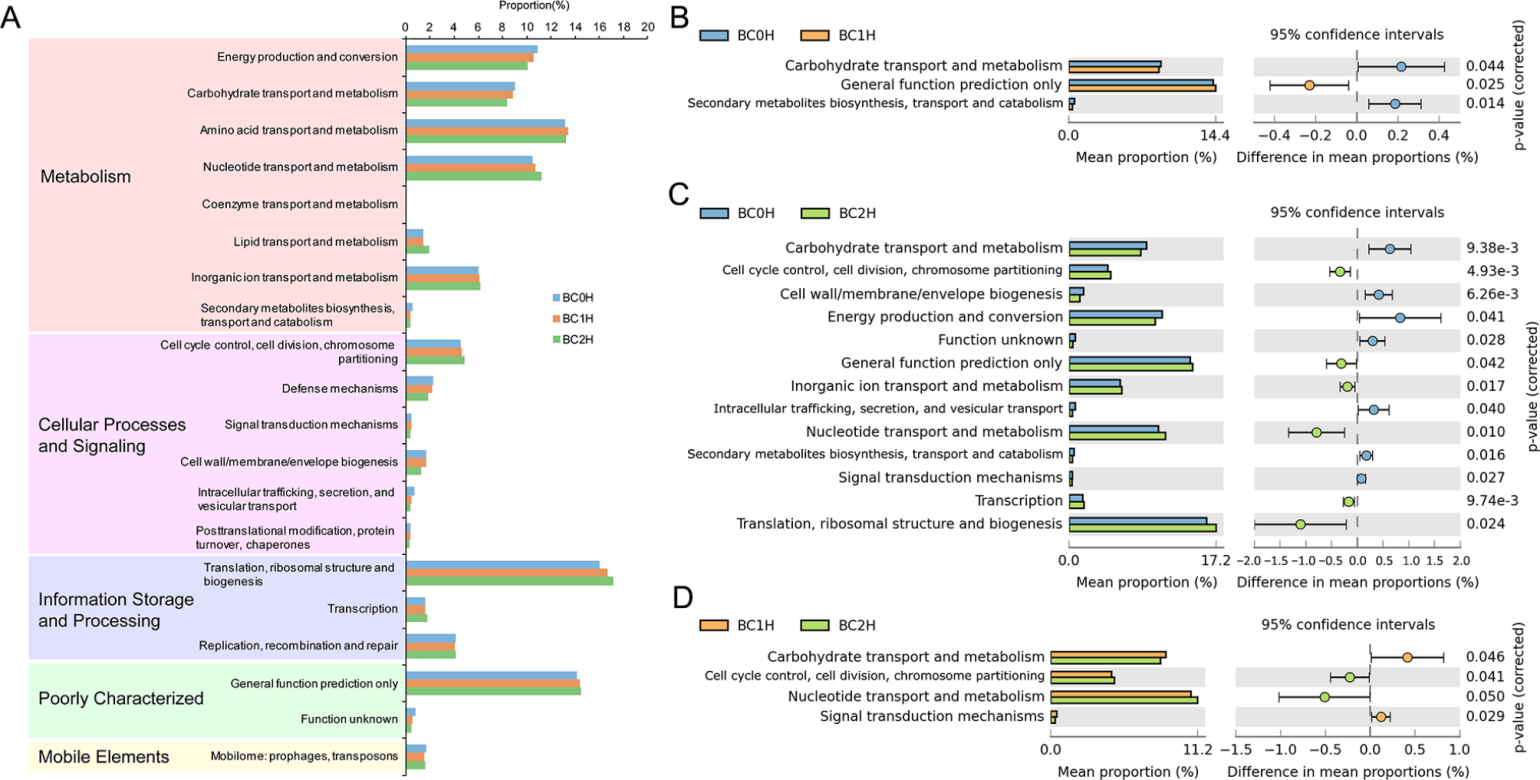
Functional predictions, clusters and comparison based on the PICRUSt analysis (**A.** General comparison among groups of fish with differing infection intensities; **B**, **C**, **D**. Pairwise comparison between groups)

Functional predictions of the results of 16s rRNA sequencing were performed using phylogenetic investigation of communities by reconstruction of unobserved states (PICRUSt). Based on the clusters of orthologous group (COG) analysis, the abundance of genes related to “energy production and conversion” and “carbohydrate transport and metabolism” categories exhibited lower abundance in infected groups (BC1H, BC2H); while the abundance of genes related to “nucleotide transport and metabolism” and “translation, ribosomal structure and biogenesis function” were relatively higher in infected groups (Fig. 3A). According to pairwise comparisons of the three groups, the abundance of genes related to “carbohydrate transport and metabolism” significantly decreased along with the intensity of infection (Fig. 3B, C and D).

### Targeted metabolic profiling

**Fig. 4:**
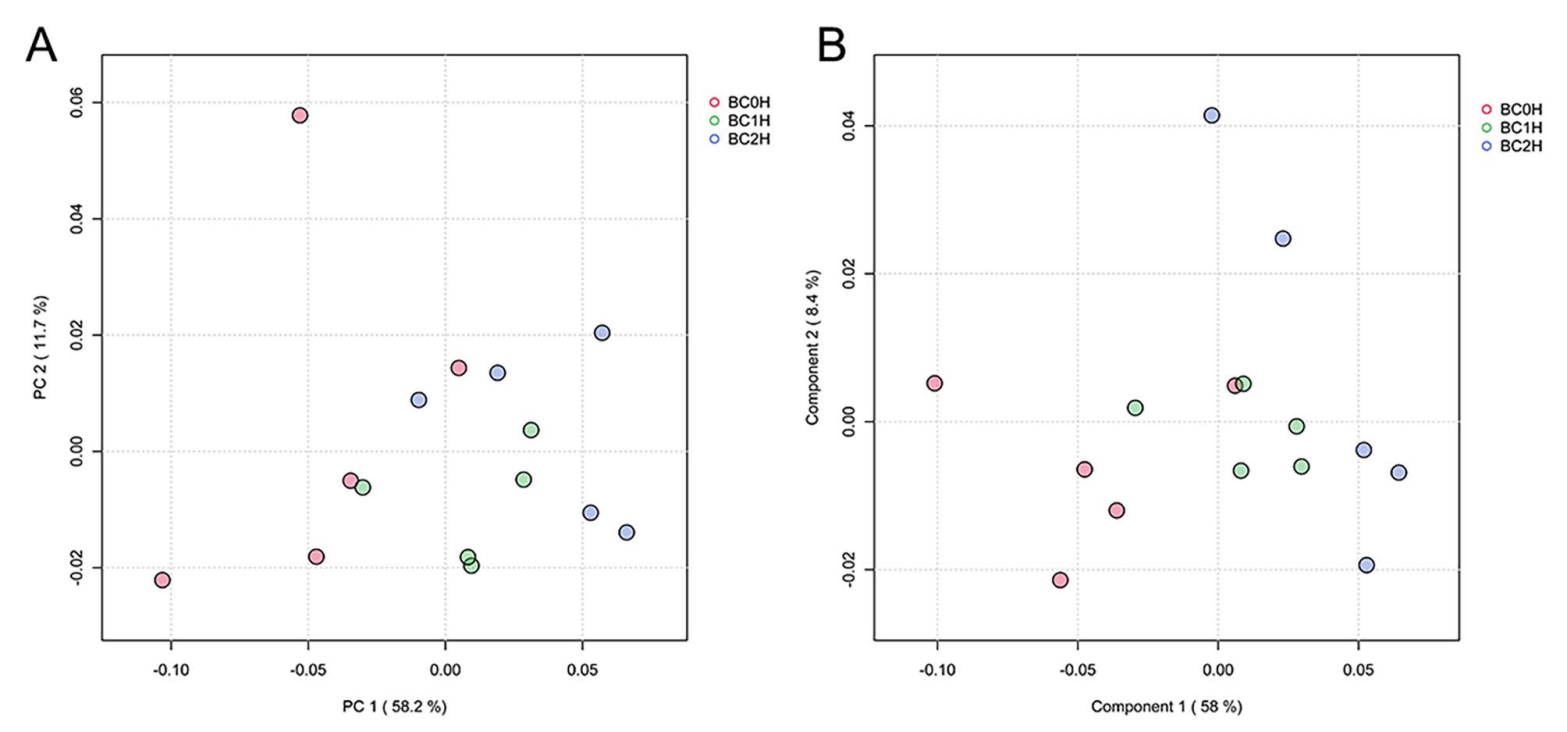
Principal component analysis (PCA, **A**) and partial least squares discrimination analysis (PLS-DA, **B**) of metabolites in the hindgut of grass carp

**Fig. 5:**
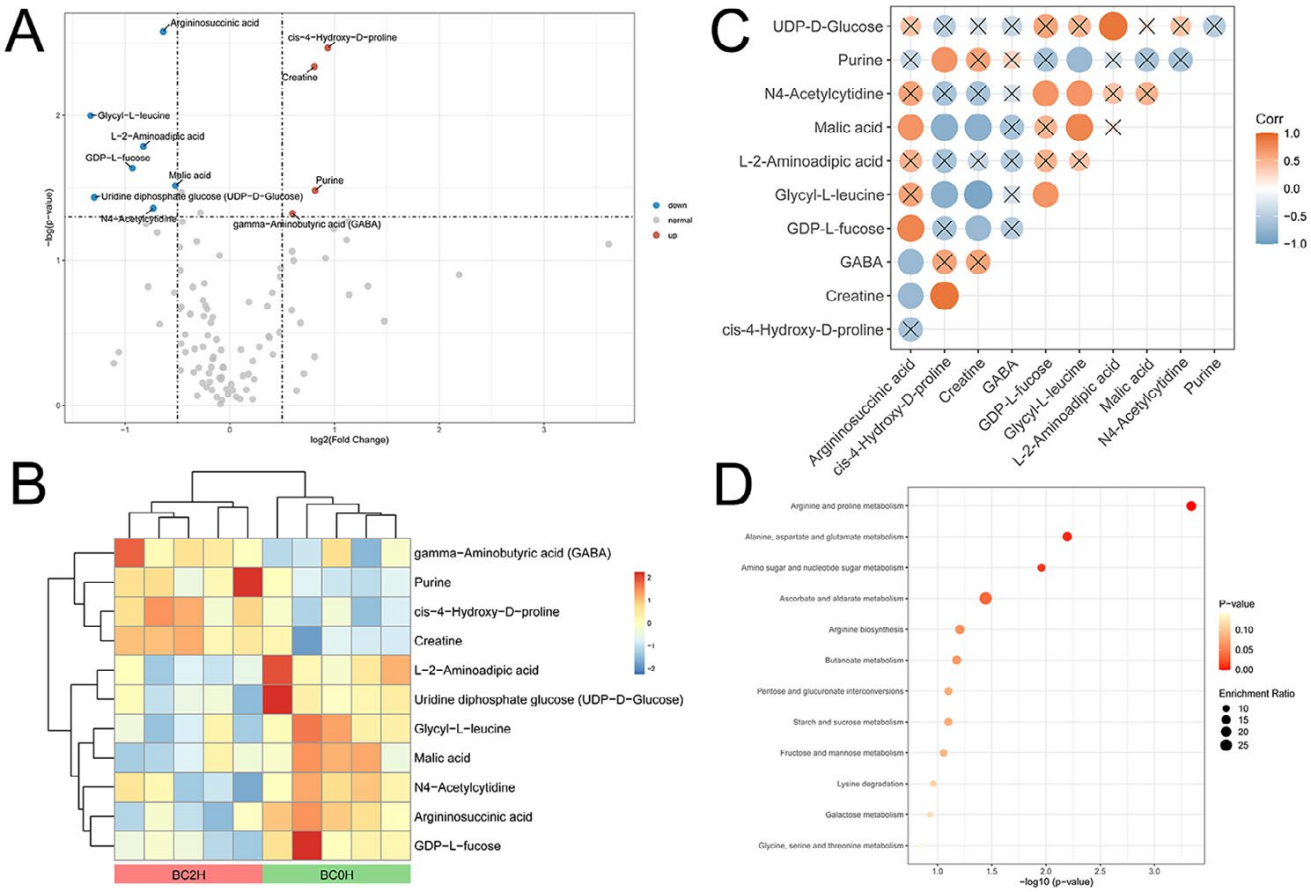
Differential metabolites between the hindgut contents of uninfected (BC0H) and high-infected (BC2H) groups identified through the targeted metabolic analysis. **(A)** Volcano plot for BC0H and BC2H groups. **(B)** Hierarchical cluster analysis of 11 differential metabolites. **(C)** Correlation analysis of 11 differential metabolites between BC0H and BC2H groups (*p* < 0.05). × represents no significant correlation between the two metabolites. **(D)** Metabolic enrichment analysis of significantly differential metabolites

Principal component analysis (PCA) revealed similar patterns of metabolites in the 3 groups (Fig. 4A). Since the PCA plot could not differentiate well, Partial least squares-discriminant analysis (PLS-DA) was used to find the differences. The score plot showed that the three groups, BC0H, BC1H and BC2H, were distinguishable (Fig. 4B). A total of 103 metabolites were detected in all three samples. Using the threshold of *p*-value < 0.05 and |log (fold change)| > 0.5, there were 11 differential metabolites between BC0H and BC2H groups, among which 4 metabolites (gamma-aminobutyric acid, purine, cis-4-hydroxy-D-proline and creatine) were up-regulated and 7 metabolites (L-2-aminoadipic acid, UDP-D-glucose, Glycyl-L-leucine, malic acid, N4-acetylcytidine, argininosuccinic acid, and GDP-L-fucose) were down-regulated in the BC2H group (Fig. 5A and B). Only 7 differential metabolites were identified between BC0H and BC1H groups (Fig. S6). Notably, creatine was the only shared differential metabolite in the two infected groups, and it accounted for a high proportion of all detected metabolites.

Furthermore, we examined the correlation among the differential metabolites (BC2H vs. BC0H). The results showed that Glycyl-L-leucine was positively correlated with GDP-L-fucose, malic acid and N4-Acetylcytidine, while it was negatively correlated with creatine, cis-4-Hydroxy-D-proline and purine (Fig. 5C). UDP-D-Glucose was positively correlated with L-2-Aminoadipic acid, and creatine was significantly correlated with cis-4-Hydroxy-D-proline (Fig. 5C). The differential metabolites were mainly involved in ascorbate and aldarate metabolism, arginine and proline metabolism (Fig. 5D).

### Co-occurrence of microbial taxa and metabolites

**Fig. 6:**
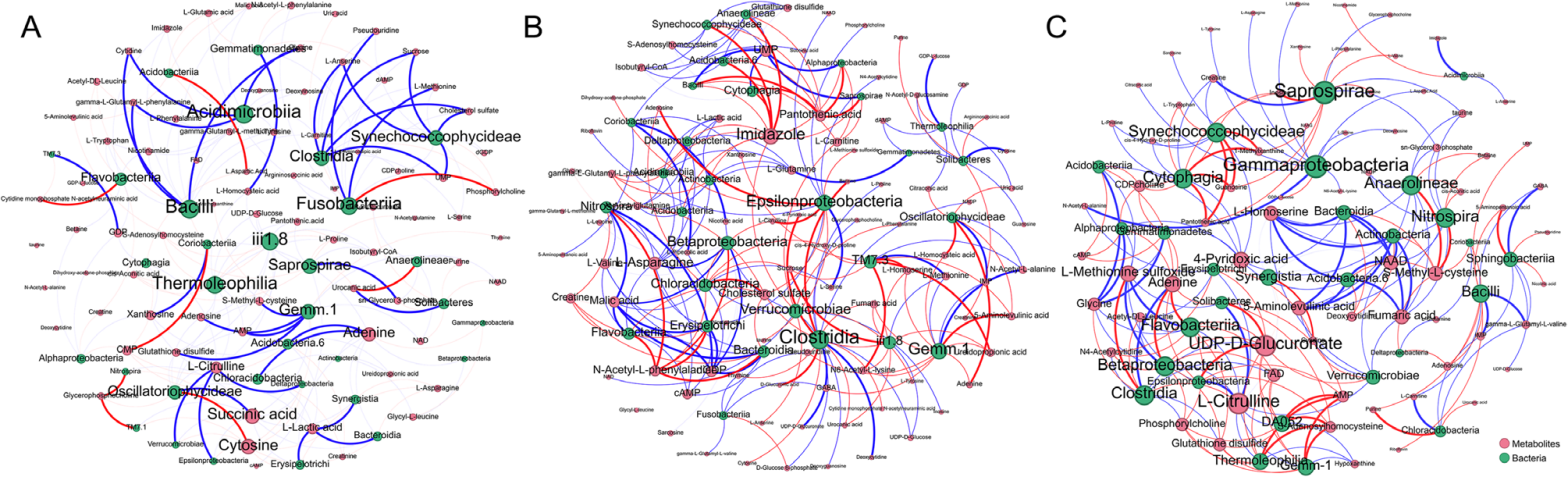
Co-occurrence networks of intestinal bacteria and metabolites in the hindgut of grass carp (**A.** BC0H group, **B.** BC1H group, **C.** BC2H group). Only significant relationships (*p* < 0.05) are shown. The blue edge indicates a positive relationship between metabolites and bacteria, and the red edge indicated a negative relationship. The width of the edge corresponds to the magnitude of the correlation between the bacteria and metabolites. The node size is proportional to the number of connections

The co-occurrence networks were constructed based on a threshold of both the correlation coefficient of > 0.8 and a *p*-value of < 0.05. Compared to the uninfected group (BC0H), the infected groups had stronger correlations between microbiota and metabolites. The three groups showed different co-occurrence patterns between microbial taxa and metabolites, and bacteria were predominant in the co-occurrence network (Fig. 6). Specifically, Bacilli, Acidimicrobiia, Clostridia and Fusobacteriia were prominent bacterial responders in the BC0H group (correlation coefficient > 0.9, *p* < 0.05), in which Bacilli and Acidimicrobiia belonged to a similar pattern correlating negatively with Cytidine, gamma-L-Glutamyl-L-phenylalanine and glycine (Fig. 6A). The correlation coefficients of Bacilli with glycine, cytidine and gamma-L-glutamyl-L-phenylalanine were about 1.0 (*p* < 0.01, Fig. S7A). In the BC1H group, Clostridia and Epsilonproteobacteria were two core bacterial responders, and many metabolites were associated with them (Fig. 6B). In the BC2H group, Gammaproteobacteria and Saprospirae were two prominent bacteria responders, followed by Betaproteobacteria, Flavobacteriia, Cytophagia, and Anaerolineae. Additionally, UDP-D-Glucuronate and L-Citrulline were the two prominent metabolite responders that were correlated with Clostridia, Betaproteobacteria and Flavobacteria (Fig. 6C, Fig. S7B).

## Discussion

The associations between microbiota and parasites have been increasingly investigated in various animal hosts in recent years [22–24]. There is evidence emerging that this relationship can influence host health, making the hosts more susceptible, tolerant, or resistant to parasites [17, 25, 26]. The introduction of a parasite can lead to disruptions in microbial composition and diversity [27], moreover, the growth and proliferation of the parasite might also be linked to the associated bacterial microbiome [20, 28]. Thus, the tripartite relationship among the microbiota, parasite and host is complex, with difficult-to-predict outcomes.

Parasite-bacteria interactions may be driven by predation, competition, or other factors. For example, protist predators have an important role in the top-down control of microbial communities [29–31]. *Balantidium* species have one oval cytostome and several food vacuoles containing the intestinal bacteria of hosts [32, 33]. We also found that there is a large proportion of carbohydrate-active enzyme genes encoding carbohydrate-binding module 50 (CBM50) with an affinity for peptidoglycan in the *B. ctenopharyngodoni* genome (unpublished data). Thus, *Balantidium* species are also bacterivorous (preying on intestinal bacteria).

### ***Balantidium ctenopharyngodoni*** alters the diversity and abundance of intestinal microbiota

Disruption of intestinal microbiota has been described in different intestinal inflammatory diseases. Healthier individuals generally harbor greater microbial alpha diversity [18, 34]. Although there were no significant differences in alpha diversity among the three groups, a slight increase was observed in the infected samples.

Firmicutes, Proteobacteria, Bacteroidetes and Actinobacteria were the predominant microbiota in all groups. Among these, three are considered to be the core microbiota in the grass carp intestine [11]. Specific gut microbiota patterns have been shown to be linked to the colonization with common parasitic protists [35]. An increase in the relative abundance of Enterobacteriaceae correlates positively with gastrointestinal or systemic inflammation in humans [36, 37]. There was a higher abundance of Gammaproteobacteria (family Enterobacteriaceae) in the patients infected with *Blastocystis* ST7, which is considered to be a pathogenic subtype of *Blastocystis* [38, 39]. However, a lower abundance of Enterobacteriaceae and a higher abundance of Clostridia were observed in *Blastocystis* subtypes with lower pathogenicity [14, 35, 40]. Similar trends in the composition of gut bacteria in response to a *Giardia* infection may give an explanation of the protective effect of *Giardia* against moderate to severe diarrhea [41, 42]. Besides, a lower abundance of Clostridiaceae and a higher abundance of Enterobacteriaceae were reported in diarrheal piglets infected with another balantidia species *Balantioides coli* [43]. In grass carp, infection with *B. ctenopharyngodoni* also seemed to be associated with changes in specific taxa, including a decrease in the relative abundance of the family Enterobacteriaceae (class Gammaproteobacteria) and an increase in the relative abundance of the genus *Clostridium* (class Clostridia). From this perspective, *B. ctenopharyngodoni* can therefore be regarded as a low pathogenic intestinal ciliate.

### ***Balantidium ctenopharyngodoni*** can reduce the relative abundance of pathogenic bacteria

The intestinal inflammation of grass carp, induced by the pathogen *Aeromonas hydrophila*, is regarded as one of the most frequently occurring diseases in culture ponds [44]. *Citrobacter* species are conditionally pathogenic bacteria in fish and the human intestine, and they are responsible for causing enteritis and other infections [45, 46]. In our study, however, the genera *Aeromonas* and *Citrobacter* were significantly less abundant in grass carp infected with *B. ctenopharyngodoni* than in uninfected grass carp. These results indicated that *B. ctenopharyngodoni* might suppress the abundance of pathogenic bacteria to maintain intestinal homeostasis.

### Infection with ***B. ctenopharyngodoni*** may also confer positive benefits on the intestine of grass carp

In general, parasite-induced changes to the gut microbiome can modulate the host’s metabolism, so colonization with parasites is associated not only with alterations in the intestinal microbiota but also with changes in host metabolites [35, 47, 48]. In this study, we identified the metabolites present in the hindgut of grass carp, and assessed the interactions between metabolites and microbiota in the infected and uninfected groups. Creatine was enriched in *B. ctenopharyngodoni*-infected groups (BC1H and BC2H), accounting for quite a large proportion of the detected metabolites (12.7% and 15.2%, respectively). It has been reported that creatine could regulate intestinal epithelial integrity and barrier function, thus producing anti-inflammatory effects [49, 50]. Inflammatory bowel disease (IBD) is a chronic immune-mediated intestinal disease that develops as a result of interactions between environmental, microbial, and immune factors in a host [51]. Since IBD is hard to be cured completely, creatine is a good adjuvant candidate for alleviating the symptoms of IBD [49]. Besides, gut microbiota can express specific enzymes (e.g. creatinase) that mediate creatine breakdown [52]. Thus, the high content of creatine in *B. ctenopharyngodoni*-infected groups indicated that *B. ctenopharyngodoni* might further influence the metabolic breakdown of creatine by regulating the composition of intestinal bacteria, and thereby positively affecting the host intestine.

## Conclusions

*Balantidium ctenopharyngodoni* is considered as a commensal organism in the hindgut of grass carp, it does not cause severe intestinal inflammation in most cases, but it may aggravate the symptoms when its host suffers from enteritis [53, 54]. In our study, all the grass carp and their intestines exhibited a normal appearance. The changes of relative abundance in specific bacterial taxa (e.g. decreased relative abundance of Enterobacteriaceae and increased relative abundance of *Clostridium*) followed similar trends to those in gut microbiota colonized by protists with low pathogenicity. Important pathogens like *Aeromonas* and *Citrobacter* showed lower abundance in *B. ctenopharyngodoni*-infected grass carp. Creatine was significantly enriched in the infected groups, which may have positive effects on the host intestine. Taken together, our results showed that *B. ctenopharyngodoni* may have some beneficial effects on the intestinal microbiota and metabolites in grass carp.

## Methods

### Sample collection

Grass carp fed on ryegrass and commercial feed were captured (n = 32) using a cast net from an aquaculture pond in Honghu City, Hubei province, China (30.07°N, 113.75°E). Then, they were quickly transported to the laboratory, where the abdominal cavity was opened after anesthetization using MS-222. The whole intestine was taken out, and the hindgut (from the last bend to the anus) was divided and opened under aseptic conditions. The infection intensity of *B. ctenopharyngodoni* was observed and enumerated using the direct microscopic counts. The samples were grouped according to the intensity: one group (BC0H) comprised uninfected samples, in which we observed no *B. ctenopharyngodoni*; another group (BC1H) comprised low-intensity samplesexhibiting 0-10^3^*B. ctenopharyngodoni* individuals/fish; and the third group (BC2H) comprised high-intensity samples exhibiting more than 10^3^ individuals/fish (Fig. S8). The contents of hindgut were collected into sterile tubes, frozen immediately in liquid nitrogen, and stored at -80 °C for subsequent treatments.

### Bacterial DNA extraction, amplification and high-throughput sequencing

Approximately 200 mg of intestinal content was used to extract total genomic DNA via QIAamp® DNA Stool Mini Kit (Qiagen, Germany) according to the manufacturer’s instructions. The concentration of extracted DNA was measured with a NanoDrop 8000 spectrophotometer (Thermo Fisher Scientific, USA). The 16s rRNA V3-V4 region was amplified by PCR using a forward primer with a unique barcode (5’-CCTACGGGNGGCWGCAG-3’) and a reverse primer (5’-GACTACHVGGGTATCTAATCC-3’) [55]. The reactions were performed with an initial denaturation step at 94 °C for 5 min, followed by 25 cycles of 94 °C for 30 s, 55 °C for 30 s and 72 °C for 90 s, and then a final extension step at 72 °C for 10 min. The PCR products were separated by 2% agarose gel electrophoresis and purified using a DNA Gel Extraction Kit (Aidlad Biotech, Beijing, China). The concentration of the purified DNA was determined using a NanoDrop 8000 spectrophotometer. Equal amounts of all samples were pooled together for library construction, and sequencing was performed on an Illumina Hiseq 2500 platform.

### Sequence data processing

Raw paired-end reads were processed using the QIIME 2 pipeline (version 2021.4.0) [56]. After trimming low-quality bases from de-multiplexed paired-end reads, the processed reads were trimmed, de-noised, and merged using DADA2 plugin incorporated in QIIME 2. Thereafter, chimera sequences were removed, and the generated amplicon sequence variants (ASVs) were summarized, and used to generate a sequence table. The ASVs were classified and taxonomically assigned using the feature classifier in QIIME2 against the Greengenes database (13_8). Then, ASVs derived from mitochondria and chloroplasts were removed. The sequence table was then rarefied to the same sequencing depth (45,000) in all samples according to rarefaction curves. Alpha diversity was evaluated by species richness (ACE and Chao1 indices) and diversity (Shannon and Simpson indices). Permutational multivariate analysis of variance (PERMANOVA) was performed to analyze the statistical significance between two groups based on a weighted UniFrac distance matrix. The principal coordinates analysis (PCoA) plot was also generated based on the distance matrix generated from a weighted UniFrac phylogenetic method. The PICRUSt was used to predict the functional clusters of each group. Linear discriminant analysis effect size (LEfSe) was performed online using the Galaxy workflow framework (http://galaxy.biobakery.org/).

### Metabolite extraction and UHPLC-MRM-MS/MS analysis

After 25 mg of each sample was taken and placed into an Eppendorf tube, 500 µl of extraction solution (acetonitrile: methanol: water = 2: 2: 1) was added to each tube. The samples were swirled for 30s, then homogenized at 45 Hz for 4 min, and ultrasound-treated for 5 min with the ice water bath. After the homogenization and ultrasound treatment were repeated three times, the samples were incubated at -40 °C for 1 h to precipitate proteins. The samples were centrifuged at 12,000 rpm for 15 min at 4 °C, and then the supernatants (about 400 µl) were transferred into new Eppendorf tubes and dried in a vacuum concentrator without heating. The dried samples were reconstituted in 100 µl of extraction solution (acetonitrile: water = 1: 1) for 10 min in the ice-water bath. The samples were vortexed for 30 s, sonicated for 10 min, and centrifuged for 15 min at 13,000 rpm, at 4 °C. Finally, 75 µl of supernatant was transferred into a fresh glass vial for LC/MS analysis. The quality control sample was the mixture of an equal aliquot of supernatants from all of the samples.

LC-MS/MS analyses were performed on a UHPLC system (Agilent 1290 Infinity, Agilent Technologies) equipped with a UPLC BEH Amide column (2.1 × 100 mm, 1.7 μm, Waters). The mobile phase consisted of 25 mM ammonium acetate and 25 mM ammonia hydroxide in water (pH = 9.75) (A) and acetonitrile (B) was carried with the elution gradient and flow rate of 0.5 ml/min. The column temperature was set at 25 °C, the sample-plate temperature was 4 °C, and the injected volume was 2 µl. An Agilent 6495 triple quadrupole mass spectrometer (Agilent Technologies) with an electrospray interface and multiple reaction monitoring modes was used for the assay development. The parameters of the typical ion source were as follows, capillary voltage = + 3000/-2500 V, gas (N2) flow = 16 L/min, gas (N2) temperature = 170 °C, sheath gas flow = 12 L/min, sheath gas (N2) temperature = 350 °C, nebulizer = 40 psi. Data were collected and processed with Agilent MassHunter Work Station software (Agilent Technologies). Differential metabolites were analyzed using a log (fold change) of 0.5 and a t-test threshold of 0.05. Principal component analysis (PCA) and partial least squares discrimination analysis (PLS-DA) were performed on the MetaboAnalyst 5.0 platform [57].

### Combined analyses of metabolome and microbiome

Co-occurrence analyses of microbiota and metabolites were performed in Rstudio 2022.12.0 + 353 with a correlation coefficient of > 0.8 and a *p*-value of < 0.05. The Gephi software (Version 0.10.1) was used to analyze and visualize the network graphs under the Fruchterman Reingold algorithm. Correlations between metabolites and bacterial communities were also assessed by Spearman’s correlation analysis, and the graph was generated using the pheatmap package (version 1.0.12, https://cran.r-project.org/web/packages/pheatmap/index.html) in Rstudio.

### Electronic supplementary material

Below is the link to the electronic supplementary material.

Supplementary Material 1

## Data Availability

The raw sequencing data used in this study were deposited at the National Center for Biotechnology Information (NCBI) with BioProject ID PRJNA1018602 (https://www.ncbi.nlm.nih.gov/bioproject/PRJNA1018602). Other data are available from the corresponding author upon reasonable request.
