# Supplementary material for: Interactions between Balantidium ctenopharyngodoni and microbiota reveal its low pathogenicity in the hindgut of grass carp
Source: BMC Microbiol. 2024 Jan 3;24:7. doi: 10.1186/s12866-023-03154-8 (PMC10762984; doi:10.1186/s12866-023-03154-8)
Supplement: Supplementary file 1 — Supplementary Material 1 [file 12866_2023_3154_MOESM1_ESM.pdf]

Supplementary Figures and Table

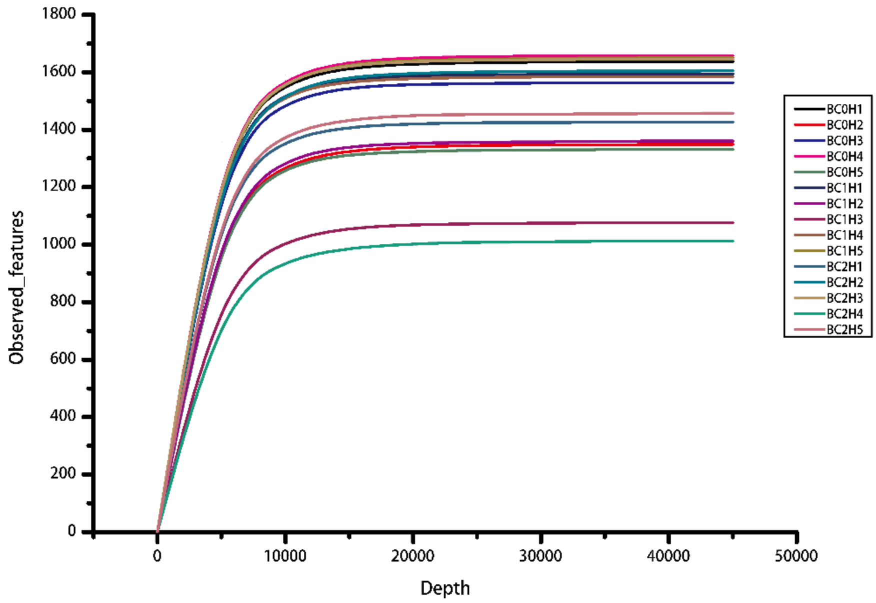

Fig. S1. Rarefaction curve based on the observed features per sample.

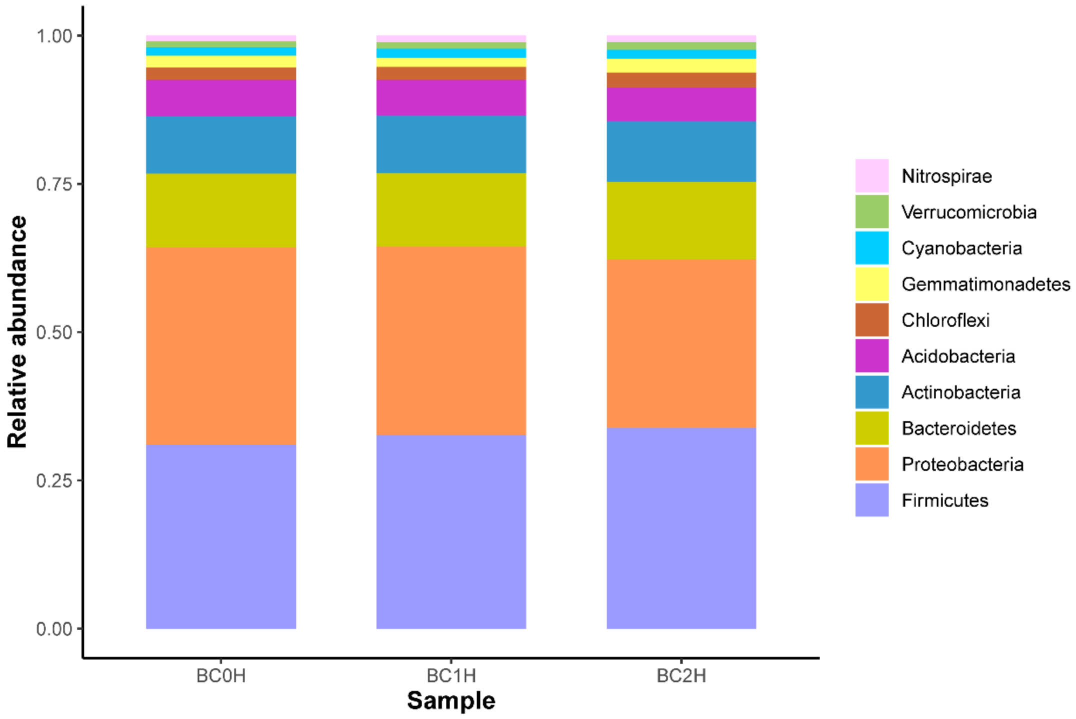

Fig. S2. Relative abundance of microbiota at the phylum level.

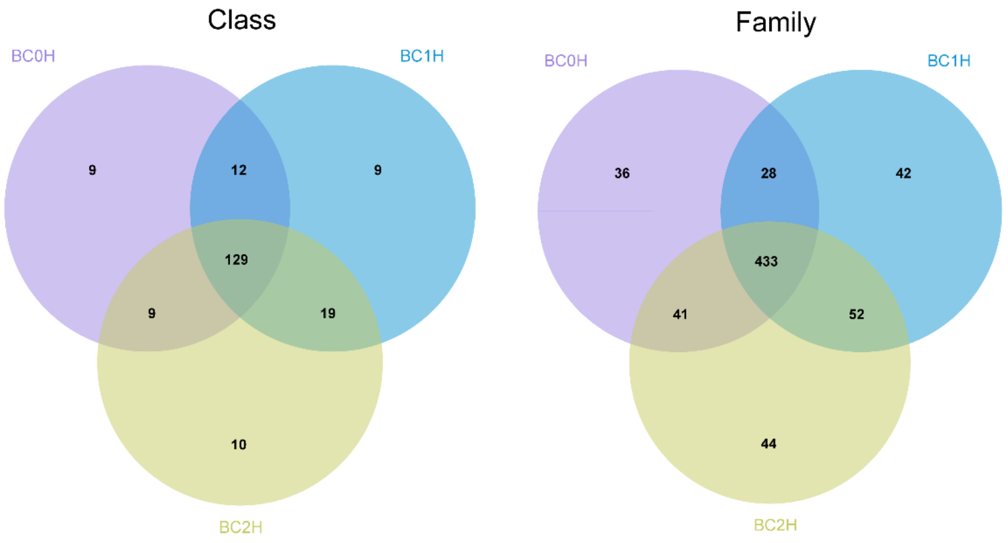

Fig. S3. Venn diagram of microbiota taxa at class and family levels.

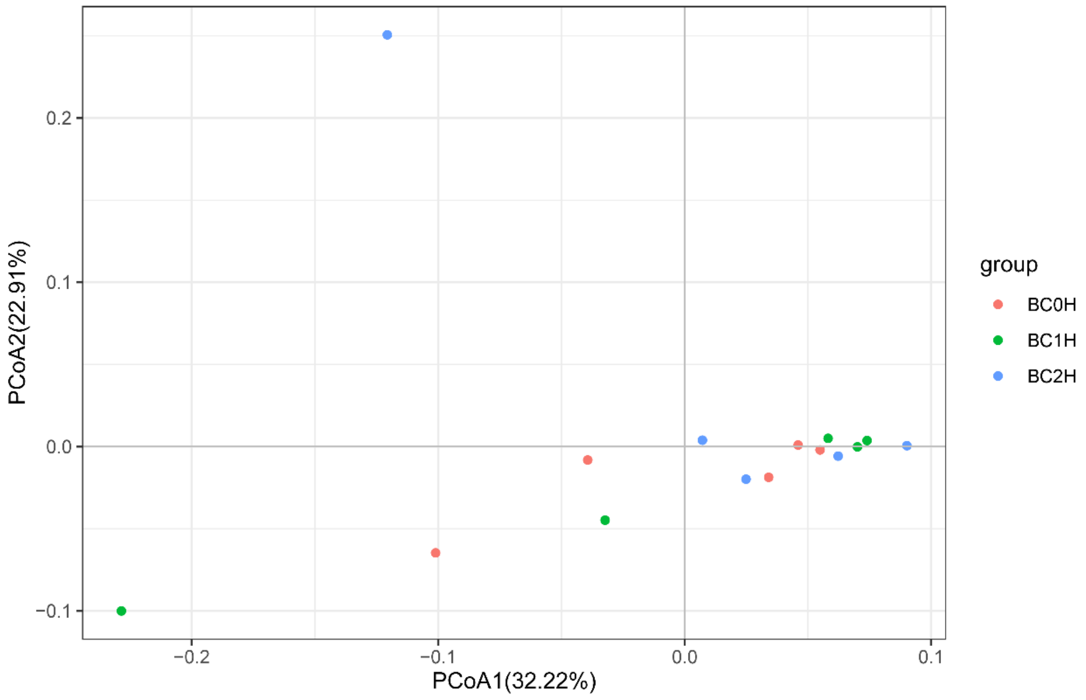

Fig. S4. Principal co-ordinates analysis based on weighted unifrac distances.

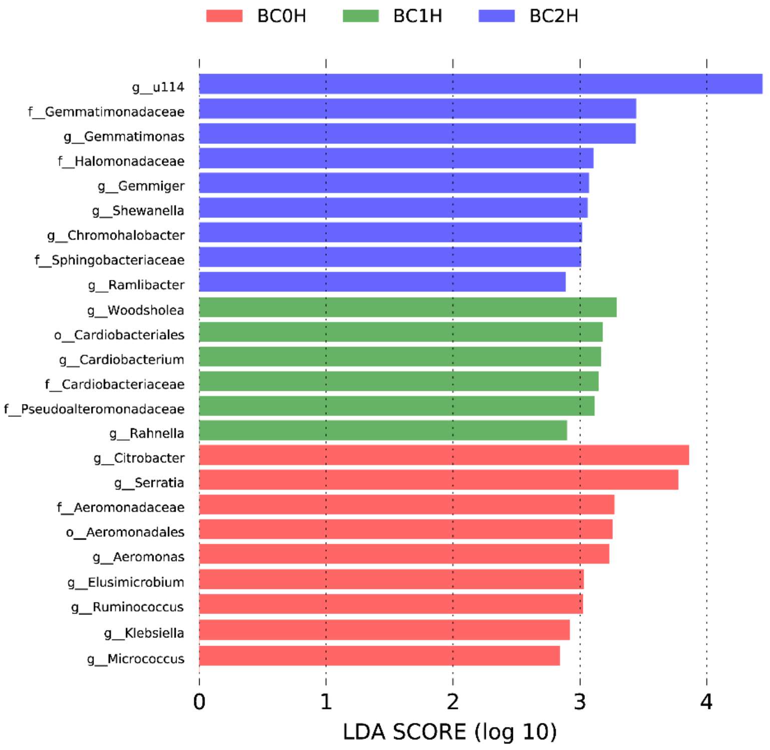

Fig. S5. The linear discriminant analysis effect size (LEfSe) analysis of the microbial abundance among three samples with an LDA threshold score of 2.5.

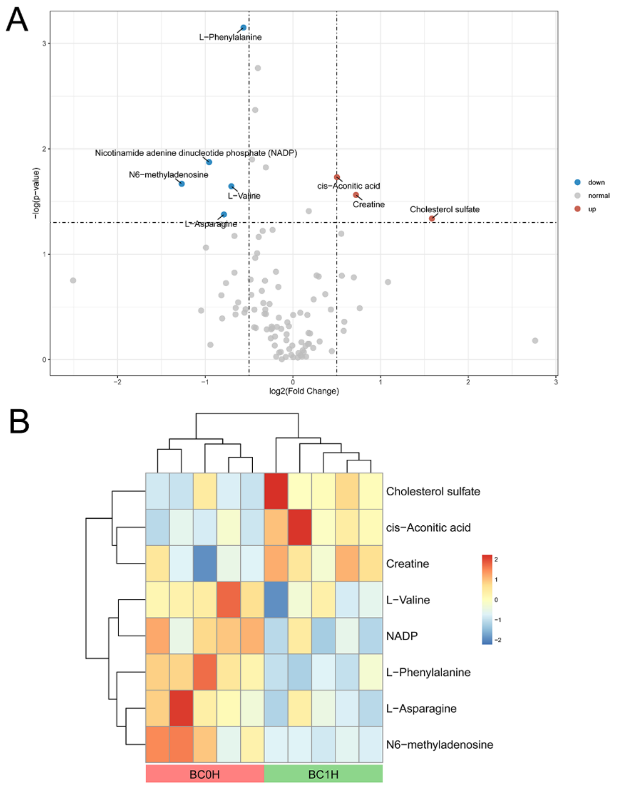

Fig. S6. Differential metabolites between BC0H and BC1H groups.

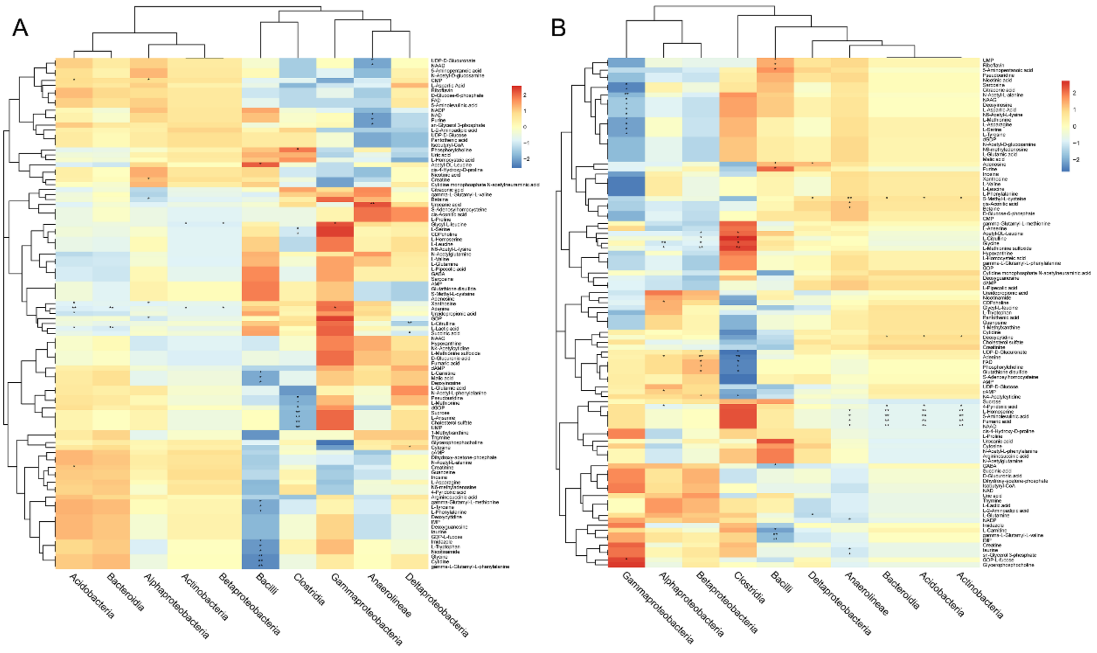

Fig. S7. Correlations between targeted-metabolic patterns and microbiota. Spearman's correlation coefficients were calculated between the top 10 enriched classes and all metabolites. Red, positive correlation. Blue, negative correlation. * indicates $p < 0.05$ and ** indicates $p < 0.01$.

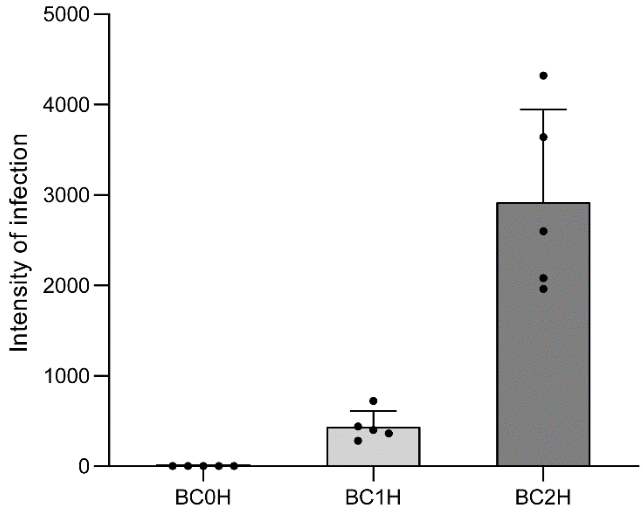

Fig. S8. The intensity of infection of *Balantidium ctenopharyngodoni* in three groups.

Table S1. Comparison of bacterial alpha diversity indices in BC0H, BC1H and BC2H groups

Index	BC0H		BC1H		BC2H		chi-squared	p-value
	Mean	SD	Mean	SD	Mean	SD		
ACE	1526.27	159.94	1563.28	127.11	1557.49	110	0.2	0.9
Chao1	1527.69	159.27	1566.08	128.84	1563.26	105.62	0.38	0.83
Shannon	9.51	0.61	9.7	0.55	9.66	0.41	1.07	0.59
Simpson	0.9942	0.01	0.9948	0.01	0.9953	0.01	0.4	0.82
